# Outcomes of military patients treated at the UK Royal Centre for Defence Medicine 2007 to 2013

**DOI:** 10.1186/cc13247

**Published:** 2014-03-17

**Authors:** AM Johnston, J Henning, D Harrison

**Affiliations:** 1 Royal Centre for Defence Medicine, Birmingham, UK; 2 James Cook University Hospital, Middlesbrough, UK; 3 ICNARC, London, UK

## Introduction

UK military personnel injured overseas are repatriated to the Royal Centre for Defence Medicine (RCDM) based at the Queen Elizabeth Hospital Birmingham (QEHB) in Birmingham UK. We report the demographics and outcomes of military patients treated on the ICU at RCDM using data from the Intensive Care National Audit and Research Centre over a 6.5-year period.

## Methods

Data on 570 admissions of 527 patients to the ICU at RCDM/ QEHB were analysed by ICNARC using standard methodology.

## Results

Some physiology and CCMDS data were missing for 175 patients. Age, sex and mortality are described in Table 1. A total of 90.9% of patients had traumatic injuries, 2.1% received CPR prior to ICU admission, 1.5% prehospital. A total of 20.6% had head, neck or spinal trauma. A total of 85.7% were transferred directly to the ICU from a military hospital overseas, others coming to the ICU following surgery at RCDM. Of the 382 patients with APACHE II score data the mean score was 11.0 (SD 4.9), probably reflecting stabilisation in military hospitals overseas or during aeromedical critical care transfer. The mean number of ICU days was Level 3: 7.6 (SD 11.6); Level 2: 2.0 (SD 2.8). A total of 70.4% of patients required advanced respiratory support for a mean of 7.5 days, and 33% required advanced cardiovascular support for a mean of 3.7 days.

**Table 1:** Case mix, demographics and outcome

*n*	570
Male (%)	97.9
Age	25.7
Trauma (%)	90.5
Died (%)	6.8
LOS	8.8
MV (%)	70.4
CRRT(%)	10.9
Neuro (%)	24.8
CV (%)	33.2

## Conclusion

The data on resource utilisation for this group of patients may inform planning of critical care support for military operations overseas.

